# Regulation of the Placental Renin-Angiotensin-Aldosterone System in Early- and Late-Onset Preeclampsia

**DOI:** 10.1134/S1607672922060011

**Published:** 2022-12-29

**Authors:** K. A. Artemieva, N. V. Nizyaeva, O. R. Baev, A. Yu. Romanov, G. V. Khlestova, M. N. Boltovskaya, A. I. Shchegolev, L. V. Kakturskiy

**Affiliations:** 1 Avtsyn Research Institute of Human Morphology, Petrovsky National Research Center of Surgery, Moscow, Russia; 2 Kulakov National Medical Research Center for Obstetrics, Gynecology, and Perinatology, Ministry of Healthcare of Russian Federation, Moscow, Russia

**Keywords:** placenta, preeclampsia, renin–angiotensin–aldosterone system, miRNA

## Abstract

Preeclampsia (PE) is one of the most dangerous complications of pregnancy, characterized by hypertension, proteinuria, and symptoms of multiple organ failure, which are detected de novo after 20 weeks of pregnancy. The renin–angiotensin–aldosterone system (RAAS) is one of the first to recognize pregnancy and is an important regulator of blood pressure. The placenta has its own RAAS, the role of which in the development of PE is not fully understood. In this work, for the first time, we characterized the expression of RAAS components and miRNAs controlling it in the placenta at various times of PE manifestation. The data obtained will allow the development of a new strategy in the future for the search for therapeutic agents for patients suffering from PE and cardiovascular diseases.

## INTRODUCTION

Preeclampsia (PE) is a pregnancy-specific syndrome characterized by de novo identified hypertension and proteinuria, as well as symptoms of multiple organ failure, developing after 20 weeks of gestation. Preeclampsia is detected in 2–8% of pregnancies and accounts for more than 70 000 maternal and 500 000 fetal/newborn deaths every year [1]. In the long term, PE contributes to the development of chronic cardiovascular and renal pathology in approximately 20% of women [2]. An important regulator of blood pressure and one of the systems that are the first to recognize pregnancy is the renin–angiotensin–aldosterone system (RAAS) [3]. During the physiological course of pregnancy, the mother’s body adapts and compensates for changing hemodynamic conditions [4]. Pathological changes in the RAAS are the main cause of superficial cytotrophoblastic invasion and impaired remodeling of the spiral arteries, which is a fundamental pathophysiological process accompanied by a series of complications. The placenta has its own RAAS, which is determined starting from 6 weeks and is most active in early pregnancy [5]. It should be noted that the biological properties of angiotensin (AT) II, the main biologically active end product of the RAAS cascade, are diverse and are not limited only to the regulation of vascular tone, which is extremely important during pregnancy. AT II binds to placental receptors and stimulates cell proliferation and migration, angiogenesis, and invasion of trophoblast cells [6]. Changes in the RAAS are not compensated in women with PE, which leads to capillary constriction, kidney damage, disturbances of water-salt metabolism, etc. [7]. Suppression of the circulatory RAAS, which is responsible for maintaining maternal homeostasis, and activation of the renal RAAS, together with the altered vascular reactivity to AT, promote the development of hypertension, kidney damage, and impaired neurohumoral control of maternal blood circulation and water-salt balance, which ultimately leads to PE [8]. It was shown that microRNAs (miR), in particular, miR-146a and miR-155, which are involved in the key pathogenetic mechanisms of preeclampsia development (hypertension, oxidative stress, inflammation, and dysregulation of the immune response), play an important role in RAAS signaling [9].

The role of the uteroplacental RAAS in the development and function of the placenta in PE is not fully understood. In this study, for the first time, we characterized the expression of RAAS components and miRs controlling it in various structures of the placenta depending on the time of PE manifestation.

The purpose of the study was to evaluate the expression of RAAS components and microRNAs controlling it in the placenta depending on the time of PE manifestation.

## MATERIALS AND METHODS

The study involved pregnant women suffering from PE and women with physiological pregnancy of the corresponding gestational age (Table 1).

**Table 1.** Number of patients in study groups

Groups	Uncomplicated pregnancy	Preeclam-psia
Total	44	40
Before 34 weeks of gestation	15	18
After 34 weeks of gestation	29	22

Criteria for inclusion in the PE group:

1. Singleton pregnancy;

2. The age of the pregnant woman is from 18 to 43 years;

3. The presence of criteria for PE (arterial hypertension (blood pressure ≥140/90 mm Hg), proteinuria (>0.3 g/L in daily urine), edema, and manifestations of multiple organ failure);

4. Delivery by caesarean section.

Patients with manifestations of pathology up to 34 weeks inclusive were classified as the early-onset PE. The groups with early- and late-onset PE corresponded to the comparison groups in gestational age.

Criteria for inclusion in both comparison groups:

1. Pregnancy that occurred in the natural cycle;

2. Delivery by caesarean section;

3. Total protein content in urine below 100 µg/mL;

4. Absence of signs of hypertensive disorders (blood pressure below 140/90 mm Hg).

For the early comparison group, the criterion was preterm operative delivery at terms of up to 34 weeks of gestation inclusive; in each of the patients, samples of placenta without signs of inflammatory infiltration (chorioamnionitis, villitis, and intervillitis) were taken under the control of histological examination.

For the late comparison group, the criterion was operative delivery for the following indications:

• The presence of a scar on the uterus after a previously performed caesarean section;

• Diseases of the eyes, retina, severe myopia (which did not affect the course of pregnancy and the placenta);

• Malposition;

• Anatomically narrow pelvis.

### Histological Examination

Fragments of placental tissue (1.5 × 1 × 0.5 cm) from patients suffering from PE and patients of the comparison groups were taken through the entire thickness of the placental disc from the marginal, paracentral, and central (including the villous chorion and the basal and chorionic plates) zones. The excised fragments were fixed in 10% buffered neutral formalin, pH 7.4 (Biovitrum, Russia) for 24 h and then embedded in paraffin. Next, they were cut into sections 4 μm thick. At least ten fragments of the placenta were examined (four from the central zone and two each from the paracentral and peripheral zones).

### Immunohistochemical Study

An immunohistochemical (IHC) study was performed using a Ventana automated immunostainer (Roche, United Kingdom) with a closed protocol for detection on paraffin sections of the placenta. The protocol included all stages of the standard procedure for IHC studies: deparaffinization of sections, unmasking of antigens, blocking of endogenous peroxidase, and incubation with primary and secondary antibodies. Visualization was performed using the Ultra View Universal DAB Detection Kit Ventana (Roche, United Kingdom). Rabbit monoclonal antibodies to angiotensinogen (1 : 300, Anti-Angiotensinogen antibody (ab276132); Abcam, United States) were used as primary antibodies, and goat anti-rabbit IgGs (Ventana, Roche, United Kingdom) were used as secondary antibodies. The manufacturer indicated a cross-reactivity of the ab276132 antibody with AT I, AT II, and angiotensinogen due to the common structure of the end fragment for all angiotensins; for this reason, the IHC reaction product was evaluated as angiotensins (AT) as a whole.

### Detection of miR in Structural Elements of the Placenta by Chromogenic in Situ Hybridization (CISH)

To detect miR-146a and miR-155, we used MiRCURY LNA™ microRNA ISH Optimization Kit (FFPE)/MiRCURY LNA™ for optimization of in situ hybridization of microRNAs (on 10% formalin-fixed and paraffin-embedded samples), including DIG-labeled LNA™ DNA probes for detection of miR-146a and miR-155 and controls: positive (5'-DIG-labeled U6 snRNA, small nuclear RNA) and negative (5'- and 3'-DIG-labeled scrambled probe). The colorimetric detection of miR was performed according to the manufacturer’s instructions (Exicon, United States, www.exicon.info).

### Estimation of IHC and CISH Staining

The intensity of the IHC reaction products and CISH was evaluated in optical density units (ODU) using a Nikon Eclipse microscope with NIS-Elements software AR3 (Czech Republic) at ×400 magnification with analysis of at least 20 fields of view. In the fields of view, there were no infarcts, calcifications, and areas of thrombosis. In the placentas of the comparison group, there were no signs of inflammatory changes. The expression values of IHC markers were multiplied by 100.

### Statistical Method

The results were statistically processed using the statistical functions of Microsoft Excel, as well as the MedCalc Statistical and BM SPSS Statistics 8 software packages. The normality of distribution of the initial data was tested using the Kolmogorov–Smirnov test. Data were presented as the median (Me) and Q1 and Q3 quartiles in the Me (Q1; Q3) format. Differences in groups were estimated using the paired Mann–Whitney *U* test. Differences were considered significant at *p* <0.05.

## RESULTS

### Immunohistochemical Examination of the Placenta

**Fig. 1.**
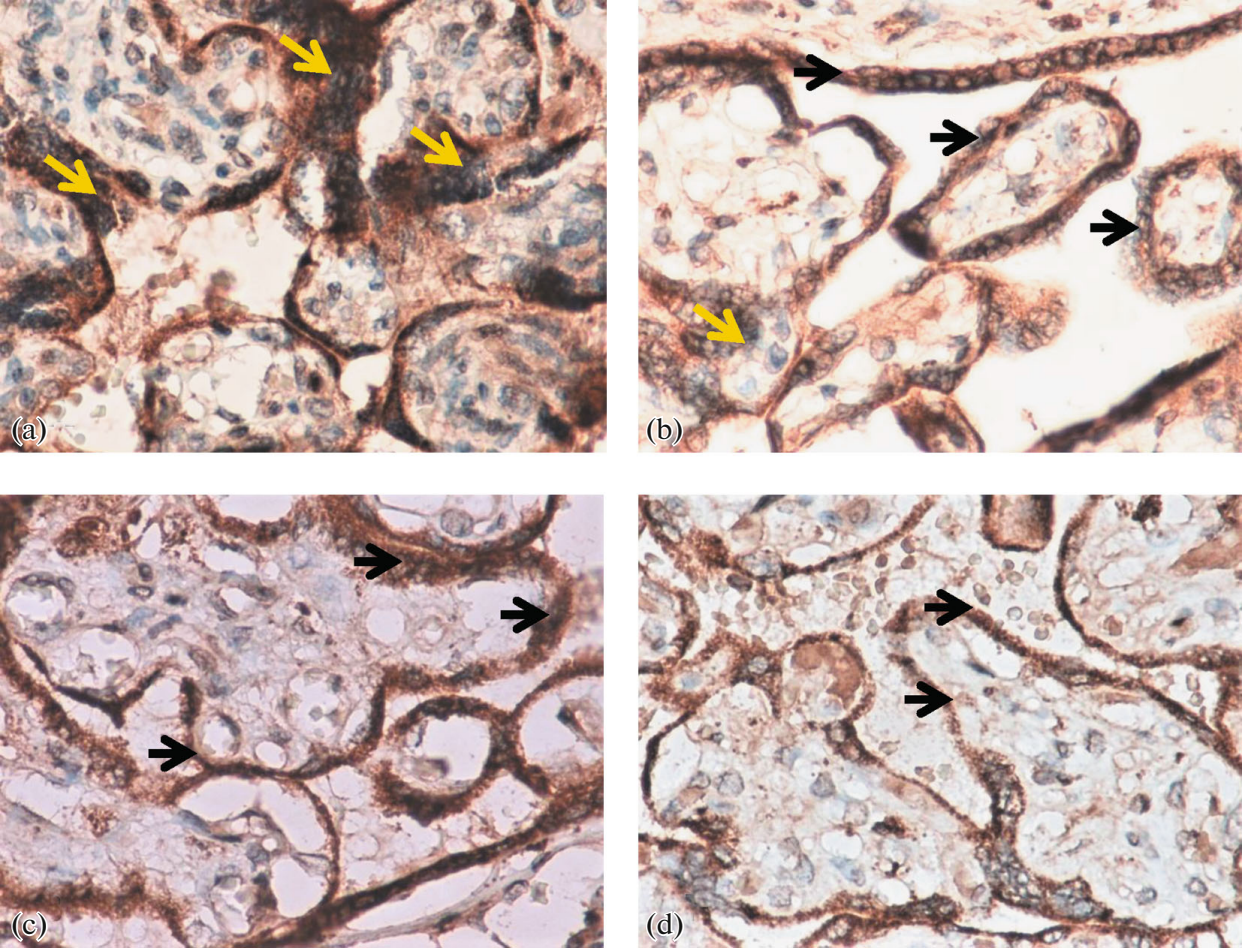
Expression of angiotensins in the placental villous tree in early- and late-onset preeclampsia (PE), ×400. (a) Early-onset PE (31–32 weeks), expression is noted in the syncytiotrophoblast (marked with a yellow arrow) and syncytial nodules (marked with a black arrow); (b) early comparison group (31 weeks); (c) late-onset PE (38 weeks); (d) late comparison group (38 weeks).

**Fig. 2.**
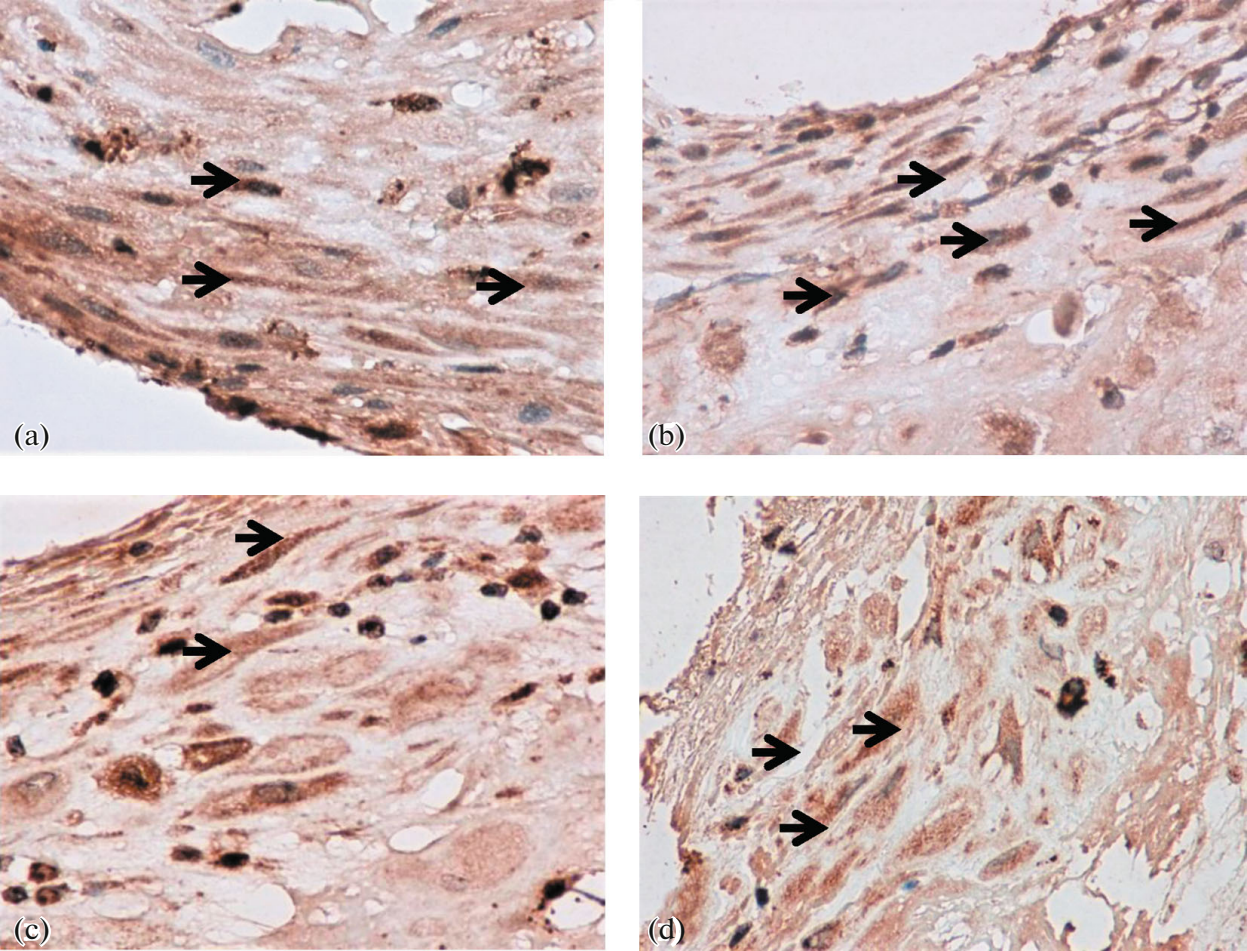
Expression of angiotensins in the decidua in early- and late-onset preeclampsia (PE), ×400. (a) Early-onset PE (32 weeks); (b) early comparison group (31 weeks); (c) late-onset PE (38 weeks); (d) late comparison group (38 weeks) (decidual cells are marked with arrows).

Analysis of the preparations revealed intense, predominantly cytoplasmic expression of AT in the cytotrophoblast (CT) and syncytiotrophoblast (SCT) of stem, intermediate, and terminal villi, syncytial nodules (SN), decidual cells, vascular endothelium, and mesenchymal cells of the villus stroma (Figs. 1, 2). It is interesting to note that weak AT expression was also detected in macrophages (MP). Both in early- and late-onset PE, the expression of the marker was higher than in the comparison groups (Fig. 3).

**Fig. 3.**
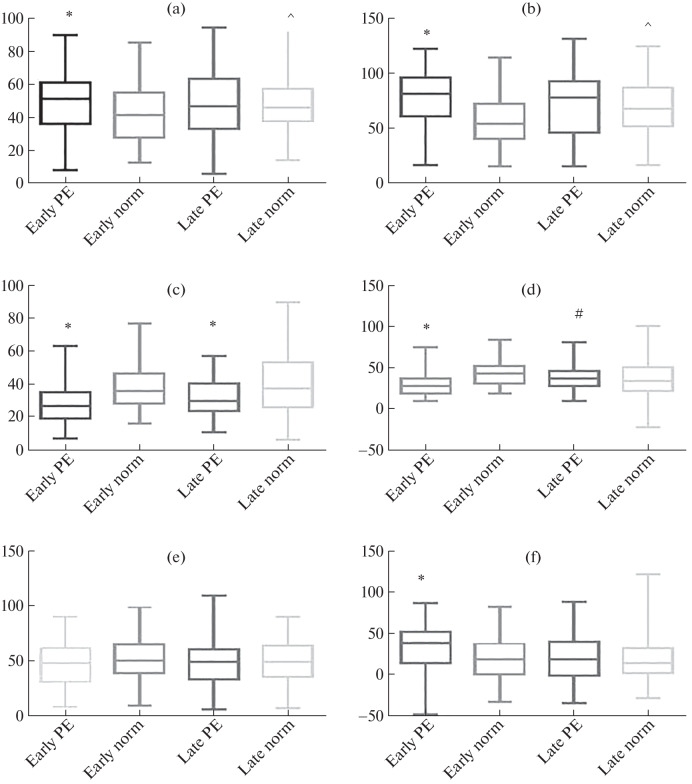
Expression of angiotensins in the structures of the placenta in early and late preeclampsia (PE): (a, b) in syncytiotrophoblast (a—membrane staining, b—cytoplasmic staining); (c, d) in the extravillous trophoblast (c—membrane staining, d—cytoplasmic staining); (e, f) in syncytial nodules (e—membrane staining, f—cytoplasmic staining); (g, h) in decidual cells (g—membrane staining, h—cytoplasmic staining); (i) cytoplasmic staining in the endothelium; (j) in fibroblast-like cells, cytoplasmic staining; (k, l) in macrophages (k—membrane staining, l—cytoplasmic staining). * Differences are significant in comparison with the control group; # differences are significant between the groups of early- and late-onset preeclampsia; ^ differences are significant between the groups of early and late norm.

**Fig. 3.**
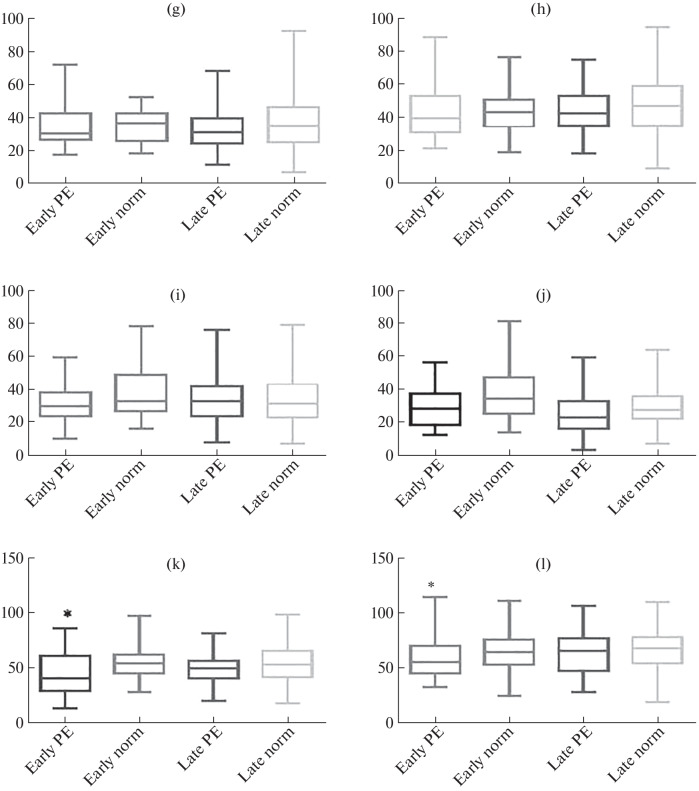
(Contd.)

### Expression of miR-146а and miR-155 in Placental Structures

During physiological pregnancy, membrane and cytoplasmic miR-146a and miR-155 staining was detected in the villous and extravillous trophoblast, syncytial nodules, and decidual cells. miR staining-155 was also detected in the MP cytoplasm. Morphometric expression was assessed in SCT and SN, where it was more pronounced. The highest miR-146a and miR-155 levels were detected at the end of uncomplicated pregnancy. Early PE was characterized by a higher level of miR-155 expression compared to the uncomplicated pregnancy group. The expression of miR-146a in the syncytiotrophoblast in the early PE was lower than in the late PE (*p* = 0.022). In the late PE, the expression levels of both miR-146a and miR-155 were significantly lower than in the comparison group (Table 2).

**Table 2.** Expression of miR-146a and miR-155 in placental villi in the early- and late-onset preeclampsia (PE), ODU (Me, Q1; Q3)

MiR	miR-146а		miR-155	
Placental structures	SN	SCT	SN	SCT
Early comparison group (ECG)	2.3 (1.63; 2.97)	1.9 (1.67; 2.13)	4.5 (3.97; 5.03)	2.5 (2.39; 2.61)
Early PE (EPE)	2.2 (1.83; 2.57)	2.0 (1.73; 2.27)	5.0 (4.59; 5.41)	4.5 (4.33; 4.68)
Late comparison group (LCG)	5.5 (4.62; 6.39)	4.4 (3.66; 5.14)	8.2 (7.72; 8.69)	8.1 (7.63; 8.58)
Late PE (LPE)	2.3 (1.96; 2.64)	2.8 (2.56; 3.04)	4.9 (4.67; 5.13)	5.1 (5.09; 5.12)
ECG–LCG	*p* = 0.003	*p* = 0.001	*p* = 0.04	*p* < 0.001
LCG–LPE	*p* = 0.001	*p* = 0.014	*p* = 0.005	*p* = 0.003
ECG–EPE	*p* = 0.62	*p* = 0.474	*p* = 0.737	*p* = 0.005
EPE–LPE	*p* = 0.81	*p* = 0.022	*p* = 0.944	*p* =0.096

It was previously believed that RAAS is associated primarily with the neuroendocrine function of the kidneys and adrenal glands. The consequences of unregulated release of RAAS components and regulators upon the trophoblast destruction include the suppression of the maternal circulatory RAAS and the activation of the intrarenal RAAS, as well as a decrease in uteroplacental blood flow. This leads to further damage to the placenta and changes in the neurohumoral control of the cardiovascular and urinary systems [10, 11], which exacerbates the hypertensive effects of the renal RAAS. The results of our study show that the placenta is an alternative renin–angiotensin system, whose structures (primarily villous and extravillous trophoblast and syncytial nodules) can produce RAAS metabolites. It was found that, in PE, AT expression is increased both in early and late manifestation of the disease.

Taking into account the fact that PE (especially severe) is associated with damage to the placenta and trophoblast, it becomes clear that desquamation and massive entry of trophoblast cells into the maternal circulation will lead to changes in the ratio of RAAS metabolites in the blood plasma, which will determine the tendency to hypertensive disorders [12]. Moreover, abnormal development, hypoxia, and damage to the placenta are associated with excessive release of exosomes and other placental particles containing angiotensins, autoantibodies to the type 1 AT II receptor (which are agonists and perform the same functions as AT II) into the maternal circulation, as well as impaired microRNA production [13]. The data obtained showed that the early-onset PE was characterized by a higher level of miR-155 expression than in the comparison group. However, miR-146a, on the contrary, showed a decrease in both early and late manifestations of PE. Despite the significant expression of AT in the endothelium of placental vessels, pronounced CISH staining of miR-146a and -155 endothelium was not observed. At the same time, it was shown that the effect of AT on human endothelial cells involves an increase in miR-146a expression and a decrease in IL-6 expression, which protects cells from its pro-inflammatory effect [14]. Probably, this distribution of miR-146a and miR-155 in the endothelium of placental vessels may serve an additional risk factor for the PE development. This assumption is consistent with the data on the downregulation of circulating miR-146a in hypertension [15]. MiR-155 plays an important role in the regulation of AT II, endothelin 1, and eNOS expression. Inhibition of miR-155 enhances NO production in endotheliocytes and acetylcholine-induced endothelium-dependent vasorelaxation [16]. Overexpression of miR-155 causes inhibition of the angiogenic inducer CYR61, a factor involved in the regulation of angiogenesis in early pregnancy, which leads to local ischemia and oxidative stress, as well as a predisposing factor in the development of PE [17]. The expression of miR-155 in the MP cytoplasm, where AT expression was also detected, is worth noting. MP express the type 1 AT receptor, which induces a shift in the MP phenotype towards the proinflammatory one (M1), thereby contributing to the RAAS-dependent hypertension [18].

In our previous work, we showed that the damaged trophoblast in PE is obviously unable to maintain miR synthesis at the required level, which leads to the formation of a vicious circle in PE and further progression of the disease [19].

Thus, in this work, we showed that the placenta has the ability to synthesize RAAS metabolites, which demonstrates the important role of the placenta in the development of hypertensive disorders. In addition, we revealed the disturbance of miR-146a and miR-155 expression in the placental structures, which, on the one hand, are involved in the regulation of the proinflammatory response, and, on the other hand, are associated with hypertensive disorders during pregnancy. The data obtained reveal new pathogenetic mechanisms in early- and late-onset PE. Further study of the role of miR may become the basis for the development of approaches to the prevention of PE and the creation of miR-based therapeutics.

## CONCLUSIONS

Thus, we demonstrated the possibility of the synthesis of the key metabolites of the renin–angiotensin–aldosterone system by the placenta, with an increase in their level in the placental structures in preeclampsia. The presented work is of fundamental importance, indicating the role of the placenta in the development of hypertensive conditions, which are associated primarily with damage to the structures of the placenta and trophoblast as an additional source of vasoconstrictors.

## References

[1] Aneman, I., Pienaar, D., Suvakov, S., et al., Mechanisms of key innate immune cells in early- and late-onset preeclampsia, *Front. Immunol.*, 2020, vol. 11, no. 1864. 10.3389/fimmu.2020.01864 PMC7462000 33013837

[2] Barbosa I.R.C., Silva W.B.M., Cerqueira G.S.G. (2015). Maternal and fetal outcome in women with hypertensive disorders of pregnancy: the impact of prenatal care. Ther. Adv. Cardiovasc. Dis.

[3] Lumbers E.R., Pringle K.G. (2014). Roles of the circulating renin-angiotensin-aldosterone system in human pregnancy. Am. J. Physiol. Regul. Integr. Comp. Physiol.

[4] Rudyuk L.A., Reshetnikova O.S. (2019). Macroscopic changes in the placenta during pregnancy complicated by congenital heart defects. Klin. Eksp. Morfol.

[5] Pringle K.G., Tadros M., Callister R. (2011). The expression and localization of the human placental prorenin/renin-angiotensin system throughout pregnancy: roles in trophoblast invasion and angiogenesis?. Placenta.

[6] Singh S., Moodley J., Khaliq O., P (2021). a narrative review of the renin-angiotensin-aldosterone system in the placenta and placental bed of HIV infected women of African ancestry with preeclampsia. Curr. Hypertens. Rep.

[7] Yang J., Shang J., Zhang S. (2013). The role of the renin-angiotensin-aldosterone system in preeclampsia: genetic polymorphisms and microRNA. J. Mol. Endocrinol.

[8] Navar L.G. (2014). Intrarenal renin-angiotensin system in regulation of glomerular function. Curr. Opin. Nephrol. Hypertens.

[9] Improta-Caria A.C., Aras M.G., Nascimento L. (2021). microRNAs regulating renin-angiotensin-aldosterone system, sympathetic nervous system and left ventricular hypertrophy in systemic arterial hypertension. Biomolecules.

[10] Fushima T., Sekimoto A., Minato T. (2016). Reduced uterine perfusion pressure (RUPP) model of preeclampsia in mice. PLoS One.

[11] Sandgren J.A., Scroggins S.M., Santillan D.A. (2015). Vasopressin: the missing link for preeclampsia?. Am. J. Physiol. Regul. Integr. Comp. Physiol.

[12] Arthurs A.L., Lumbers E.R., Pringle K.G. (2019). MicroRNA mimics that target the placental renin-angiotensin system inhibit trophoblast proliferation. Mol. Hum. Reprod.

[13] Khlestova G.V., Romanov A.Yu., Nizyaeva N.V. (2018). Dynamics of renin, angiotensin II, and angiotensin (1–7) during pregnancy and predisposition to hypertension-associated complications. Bull. Exp. Biol. Med.

[14] Gareev I.F. (2018). The role of mi-RNA in the regulation of pathophysiological mechanisms in arterial hypertension. Nauka Molodykh.

[15] Roganović J. (2021). Downregulation of microRNA-146a in diabetes, obesity and hypertension may contribute to severe COVID-19. Med. Hypotheses.

[16] Nemecz M., Alexandru N., Tanko G. (2016). Role of microRNA in endothelial dysfunction and hypertension. Curr. Hypertens. Rep.

[17] Pankiewicz K., Fijałkowska A., Issat T. (2021). Insight into the key points of preeclampsia pathophysiology: uterine artery remodeling and the role of microRNAs. Int. J. Mol. Sci.

[18] Rucker J.A., Crowley S.D. (2017). The role of macrophages in hypertension and its complications. Pflugers Arch.

[19] Nizyaeva N.V., Kulikova G.V., Nagovitsyna M.N. (2017). Expression of microRNA-146a and microRNA-155 in placental villi in early- and late-onset preeclampsia. Bull. Exp. Biol. Med.

